# Evaluation model of urban tourism competitiveness in the context of sustainable development

**DOI:** 10.3389/fpubh.2024.1396134

**Published:** 2024-06-12

**Authors:** Jingya Song, Bo Xu

**Affiliations:** ^1^School of Food Science, Henan Institute of Science and Technology, Xinxiang, Henan, China; ^2^Department of Tourism and Geographical Sciences, Baicheng Normal University, Baicheng, Jilin, China

**Keywords:** urban tourism, competitiveness, sustainable development, portfolio empowerment, Topsis

## Abstract

In the contemporary context marked by globalization and the growing prominence of sustainable development, assessing urban tourism competitiveness has emerged as a crucial research domain. This paper aims to develop a comprehensive model for evaluating city tourism competitiveness, grounded in the principles of sustainable development. The model incorporates factors such as city tourism resources, environmental considerations, economic aspects, and societal factors. This holistic approach seeks to offer valuable insights for the city tourism industry. The study conducts a thorough analysis of current research both domestically and internationally, highlighting gaps and articulating the objectives and significance of the research. Employing a machine learning-based empowerment method, the paper determines the significance of evaluation indices and utilizes the Topsis method for assessing urban tourism competitiveness. Distinguishing itself from traditional evaluation methods, this model integrates the principles of sustainable development throughout the evaluation process, with environmental, social, and economic sustainability serving as pivotal evaluation indicators. Empirical analysis involves the evaluation of tourism competitiveness for select cities, facilitating inter-city comparisons. Results from empirical studies demonstrate the model’s effectiveness in evaluating urban tourism competitiveness, providing targeted developmental recommendations for urban tourism.

## 1 Introduction

In today’s increasingly globalized global economy, urban tourism, as an important economic activity, not only plays a key role in promoting the development of local industries but also becomes an important aspect of sustainable urban development (1). The concept of sustainable development emphasizes the balance of economic, social, and environmental aspects, and the sustainability of urban tourism, as a complex system involving multiple fields, has attracted extensive attention from scholars and practitioners (2).

With the growth of population and the continuous progress of urbanization, urban tourism competitiveness has become a topic of great concern (3). Urban tourism competitiveness is directly related to the status and attractiveness of a city in the tourism market, so how to scientifically evaluate the competitiveness of urban tourism has become an urgent problem for city managers and decision-makers (4). In the context of sustainable development, the evaluation of urban tourism competitiveness should not only consider the economic benefits, but also comprehensively consider social, cultural, and environmental factors to ensure that the development of urban tourism is not only a short-term economic stimulus but also a long-term sustainable development path (5, 6).

As an important component of economic development, urban tourism not only creates employment opportunities and financial revenue for cities but also has a profound impact on urban sustainability through activities that promote culture, social interaction, and environmental protection. However, with the booming development of global tourism and the intensifying competition among cities, the enhancement of urban tourism competitiveness has become a common challenge for all cities. In this context, constructing a scientific and reasonable evaluation model of urban tourism competitiveness is of great significance for formulating reasonable urban tourism development strategies and improving the overall competitiveness of cities (7).

This paper constructs an evaluation model of urban tourism competitiveness in line with the context of sustainable development to comprehensively and scientifically assess the competitiveness of cities in the field of tourism. Through in-depth analysis of the elements of the city tourism system, aims to provide city decision-makers with more comprehensive information so that they can plan and manage the city tourism development more effectively and promote its development in a sustainable and balanced direction.

The urban tourism competitiveness evaluation model proposed in this study is based on the concept of sustainable development, focusing on multidimensional factors such as public health, environment, society, and economy. The core of the model lies in the use of machine learning technology to determine the weights of the evaluation indicators and the quantitative evaluation combined with the Topsis method, aiming to provide a scientific and comprehensive competitiveness assessment of urban tourism, to promote the sustainable development of urban tourism.

Compared with the traditional evaluation model, this model has innovations and contributions in the following aspects:

### 1.1 Sustainable development perspective

This model deeply roots the concept of sustainable development in the evaluation model, which not only focuses on the economic benefits of tourism but also emphasizes the performance of the environment, society, and public health, which helps to guide the development of urban tourism in a healthier and more sustainable direction.

### 1.2 Machine learning assignment method

Machine learning algorithms are used to determine the weights of evaluation indicators, which improves the scientific and objective nature of the evaluation process. This method can automatically learn from a large amount of data to find out the most appropriate weight allocation, thus making the evaluation results more accurate.

### 1.3 Quantitative evaluation by Topsis method

The quantitative evaluation by Topsis method can effectively deal with the decision-making problem of multiple indicators and objects, which makes the evaluation results more credible and practical.

### 1.4 Empirical analysis and recommendations

Through empirical analysis, this study evaluates the tourism competitiveness of specific cities and provides targeted development recommendations for urban tourism based on the evaluation results, which helps policymakers optimize the allocation of tourism resources and enhance the competitiveness of urban tourism.

## 2 Literature review

**Figure 1:**
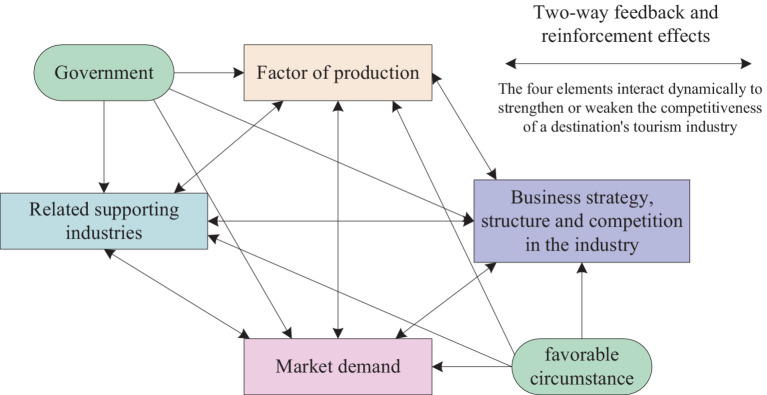
Diamond model.

Foreign research on the construction of a tourism competitiveness evaluation system started earlier (8). Starting from the late 1980s, with the emergence of Michael Porter’s competition theory and diamond model (as shown in Figure 1), quantitative research on tourism competitiveness has proliferated (9, 10). By the end of the 20th century and the beginning of the 21st century, some scholars emphasized the important role of tourism information in urban tourism competition (11). Tourists tend to choose destinations with more information (12). At the same time, the evaluation research of city tourism competitiveness expanded to many basic indicators of the city, forming a comprehensive evaluation system that emphasizes more on supporting conditions. Research on tourism competitiveness also uses various methods such as ANOVA, behavioral theory, linear regression, and other methods for quantitative evaluation of urban tourism competitiveness (13, 14). Literature (15) proposed a GIS-based model for assessing urban tourism potential. The model uses a novel hybrid modeling approach to assess the city’s tourism potential.

Domestic research on tourism competitiveness began in the 1990s. Hao Shouyi et al. conducted a preliminary study on the concept, meaning, and measurement of urban tourism competitiveness (16). Domestic research on tourism competitiveness mainly focuses on two aspects. On the one hand, it is to analyze and explore the measurement of the influencing factors of competitiveness, and on the other hand, it is based on the construction and application of the evaluation system (17). In terms of influencing factors, a considerable number of scholars use three main measurement indicators, namely, city tourism performance or status quo, city tourism development trend or potential, and city economic and social environment base, to establish a multilevel indicator evaluation system (18). The main methods are hierarchical analysis, cluster analysis, entropy value method, etc. (19–21). Literature (22) proposed a sustainability and competitiveness evaluation framework for measuring tourism destinations through data envelopment analysis. Literature (23) proposed a tourism competitiveness evaluation model for urban historical and cultural neighborhoods. The model adopts multi-source data and uses hierarchical analysis for competitiveness evaluation. Literature (24) constructed a tourism land competitiveness evaluation model based on the neural network method to reveal the pattern of rural tourism land competitiveness in Miyun District.

Although these existing studies provide rich perspectives for assessing the competitiveness of urban tourism, there is still some room for research. One of the notable spaces is that the concept of sustainable development has not yet been fully integrated into the evaluation system in a comprehensive and in-depth manner. Traditional research usually focuses on economic perspectives such as tourism resources, economic efficiency, and market size, but pays insufficient attention to the trinity of environmental, social, and economic dimensions of sustainable development.

Specifically, in terms of environmental sustainability, although some studies have begun to recognize the importance of environmental protection to tourism competitiveness, in-depth research is still needed on how to effectively integrate environmental factors into the evaluation system of urban tourism competitiveness and quantify their impact on the long-term healthy development of tourism. As for the social dimension, considerations on how tourism activities can promote community development, protect cultural heritage, enhance the level of public services, and safeguard social equity have not been adequately reflected in the established evaluation models.

In addition, another research gap is manifested in the degree of modernization and intelligence of evaluation methods. When setting the weights of evaluation indexes, many current studies still use fixed weight allocation or a method based on expert judgment, which largely ignores the great potential of big data and intelligent algorithms in revealing the deep structure and dynamic evolution of tourism competitiveness.

Given this, this paper innovatively constructs a set of urban tourism competitiveness evaluation models based on the principle of sustainable development. Based on the original evaluation of tourism resources, tourism economy, and tourism environment, the model incorporates sustainability into consideration for the first time to ensure the comprehensiveness and three-dimensionality of the evaluation system. Through the introduction of the machine learning weighting method, the model can dynamically and accurately assign reasonable weights to the evaluation indicators based on the actual data, thus effectively overcoming the subjectivity and static nature of the traditional evaluation methods in weight setting. After that, the Topsis method is used to evaluate the competitiveness of urban tourism. The model in this paper provides personalized development suggestions for the urban tourism industry in line with the principle of sustainable development, enriches the theoretical framework of urban tourism competitiveness evaluation, and highlights the importance of the model in promoting the sustainable development of the tourism industry.

## 3 State of the art

### 3.1 Importance of the concept of sustainable development for the tourism industry

**Figure 2:**
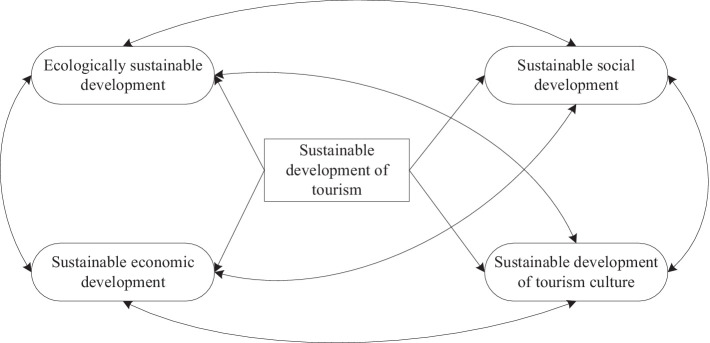
Four key dimensions for achieving sustainable tourism development.

Sustainable tourism activities include environmental, economic, social, and cultural aspects of tourism development. The concept of these four aspects of sustainable tourism development can be illustrated in Figure 2. As natural resources may be exploited in the tourism industry, tourism activities sometimes have significant impacts on the ecosystem, economy, society, and tourism culture. Thus, to prevent climate change and ecological degradation, it is important to take into account the balance of these four dimensions, so that the tourism industry can develop in the short term, while at the same time better ensuring long-term sustainable development. The principle of sustainable development should be applied to all types of tourism activities and tourism operations, and tourism enterprises should establish long-term and short-term strategies and plans.

**Figure 3:**
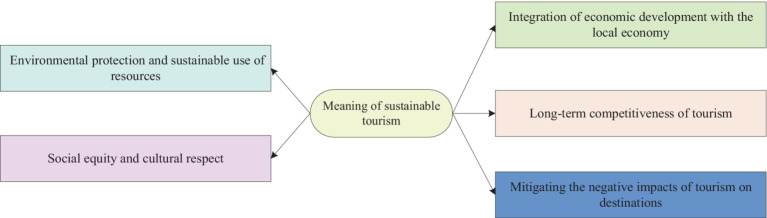
Implications of the concept of sustainable development for the tourism industry.

The concept of sustainable development has important and far-reaching significance for the tourism industry, which is mainly reflected in the following five aspects, as shown in Figure 3.

#### 3.1.1 Environmental protection and sustainable use of resources

Tourism is often dependent on the natural environment and cultural resources, which are limited. The concept of sustainable development emphasizes the protection of the natural environment, ecosystems, and cultural heritage to ensure that they can be used sustainably in the long term. By adopting environmentally friendly measures and promoting the use of renewable energy sources, tourism can reduce its negative impact on the environment and ensure the sustainability of its resources.

#### 3.1.2 Social equity and cultural respect

Sustainable development encourages the tourism industry to focus on social equity and cultural respect alongside economic development. This includes providing equitable employment opportunities, respecting the culture and traditions of local communities, and ensuring that tourism development does not negatively impact local societies. By adopting socially responsible and culturally respectful practices, tourism can contribute to socially and culturally sustainable development.

#### 3.1.3 Integration of economic development with the local economy

The sustainable development of tourism contributes to the realization of sustained economic growth and ensures its integration into the local economy. By promoting the participation of local businesses and communities in the tourism value chain, tourism can become an engine of economic development, leading to the development of local employment and industry. This helps to reduce poverty and improve the standard of living of the local population.

#### 3.1.4 Long-term competitiveness of tourism

Sustainable development is a key factor in the long-term competitiveness of the tourism industry. As society’s focus on sustainability issues continues to grow, so do consumer demands for environmental protection, social responsibility, and cultural respect. Adopting sustainable practices can help improve corporate image, enhance brand value, and attract more tourists and investment. Therefore, sustainable development has become a strategic advantage in the highly competitive tourism market.

#### 3.1.5 Mitigating the negative impacts of tourism on destinations

The concept of sustainable development emphasizes minimizing negative impacts on destinations, including environmental damage, overexploitation, and cultural erosion. Through sustainable planning and management, the tourism industry can ensure that tourist visits minimize the impact of the destination, thereby protecting its unique natural and cultural resources.

Taken together, the concept of sustainable development for tourism implies balancing and harmonizing the economic, social, and environmental dimensions. Such a holistic approach contributes to a healthier, more stable, and sustainable tourism model.

### 3.2 Machine learning algorithms

#### 3.2.1 Logistic regression

Logistic regression is: widely used in the fields of tourism suitability evaluation and tourism competitiveness evaluation (25, 26). Compared with general regression models, the advantage of this model is that its independent variables do not need to satisfy the normal distribution and can be discrete or continuous. In this model, the correlation between variables can be expressed as Equation 1:


(1) $$u=\frac{1}{1+e^{-k}}$$

Where: u represents the fitness probability of the event occurring, floating in the interval [0, 1] on the Sigmond function curve. k represent a linear combination of the independent variables.

LR is built with the “Logistic Regression” module of the Sklearn library in Python 3.8.

#### 3.2.2 Support vector machines

SVM is a learning method to solve nonlinear linkage and high-dimensional problems by constructing an optimal plane (27, 28). With a solid statistical theoretical foundation and a concise mathematical model, it has a unique advantage in solving small-sample identification problems and is widely used in the fields of tourism demand forecasting, tourism potential evaluation, and tourism sentiment analysis. The objective function *L* of the optimal solution of this model is Equation 2:


(2) $$L\left(m,c,g\right)=\frac{1}{2}m^{T}m+\overset{u}{\underset{x=1}{\sum }}g_{x}\left[1-j_{x}\left(m^{T}i_{x}+c\right)\right]$$

Where: m is a set of vectors representing the weights. $m^{T}$ is a column vector of this set of weights. u represents the total number of samples. $g_{x}$ represents the Lagrange multiplier. $j_{x}$ represents the actual value of the *x*th sample. $i_{x}$ represents the eigenvalue of the *x*th sample. c is a constant.

The construction of SVMs is done using the “SVC” module of the Sklearn library in Python 3.8.

#### 3.2.3 Random forest

RF is a Bagging integrated learning algorithm with decision trees as base classifiers (29), which has achieved good research results in the fields of tourism preference prediction and tourism land suitability evaluation. The model contains multiple decision tree submodels, and the classification results of the model are determined by the classification results of each decision tree. Therefore, it has high stability and expandability. The basic formula of the model is:


(3) $$f\left(i\right)=\frac{1}{H}\overset{H}{\underset{h=1}{\sum }}N_{h}\left(i;\theta_{h}\right)$$

Where in Equation 3: $f\left(i\right)$ is the output. i is the independent variable. h is the number of trees in the decision tree. $\theta_{h}$ is the parameter vector of the bth tree. $N_{h}\left(i;\theta_{h}\right)$ is the CART algorithm for constructing the decision tree.

The construction of RF is realized in Python 3.8 using the “Random Forest Classifier” module of the Sklearn library.

#### 3.2.4 Extreme gradient boosting tree

XGB is a Boosting integrated learning model based on iterative operations of weak classifiers (30). Since its proposal in 2017, XGB has been widely used in the fields of tourism destination image evaluation and tourism traffic trend prediction with its advantages of high accuracy and scalability. Its weak classifiers are both tree and linear models, and it can utilize the central processor to perform complex multi-threaded parallel operations, thus improving the model prediction accuracy, stability, and computational efficiency.

XGB is built in Python 3.8 using the “XGBClassifier” module of the Xgboost library.

The challenge of sustainable tourism is how to enhance the benefits of tourism by moving in the right direction, while at the same time requiring the ability to mitigate the negative impacts of other aspects. In this paper, machine learning and the Topsis method are combined to construct a city tourism competitiveness model. The model can give the strengths and weaknesses of different cities in the field of tourism and provide targeted development suggestions for sustainable urban tourism.

## 4 Methodology

### 4.1 Technical routes

**Figure 4:**
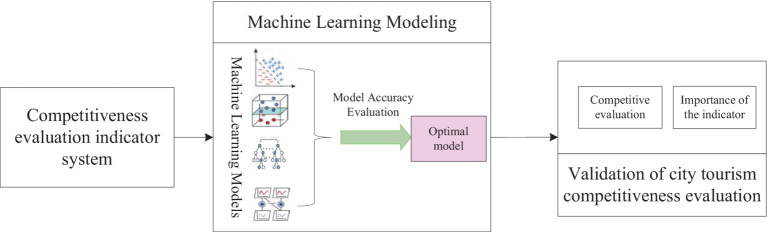
Technical route.

The technical route of this paper is shown in Figure 4, including the construction of an indicator system, machine learning modeling, and evaluation of urban tourism competitiveness in three parts: (1) Establish the evaluation indicator system of urban tourism competitiveness around the perspective of resources, natural environment, and other factors, form the indicator factor data set, and then calculate the indicator value of each evaluation unit. (2) Substitute the index data into each machine learning model for training, compare the performance evaluation criteria of each model, and then select the optimal model. (3) The optimal model is used to identify the competitiveness of city tourism and output the importance degree of evaluation indexes. Construct a Topsis assessment model to evaluate urban tourism competitiveness.

In selecting machine learning algorithms, this paper considers the interpretability, accuracy, generalization ability, and computational efficiency of the model. In this paper, the random forest model was chosen for assignment because it is insensitive to noise and outliers in the data and can provide importance scores for variables. In the evaluation of urban tourism competitiveness, random forest can consider multiple indicators comprehensively and reduce the impact of outliers of individual indicators.

In constructing the indicator system, this paper follows the principles of comprehensiveness, representativeness, and operability to ensure that the indicators can comprehensively cover all aspects of urban tourism and accurately reflect the key elements of urban tourism competitiveness.

Compared with traditional evaluation methods, the evaluation model based on machine learning has stronger data processing capabilities, captures the nonlinear relationship between variables, has better generalization capabilities, and can automatically adjust the weights according to new data and feedback.

In the context of sustainable development, this model not only considers economic factors but also incorporates environmental and social sustainability indicators, which is an important improvement over traditional evaluation methods. By taking these factors into account, this model can evaluate the competitiveness of urban tourism more comprehensively and provide targeted development suggestions for urban tourism.

### 4.2 Competitiveness evaluation indicator system

The selection and construction of evaluation indicator of urban tourism competitiveness directly affect the objectivity and scientificity of the study, therefore, when measuring the level of urban tourism competitiveness, it is usually necessary to establish an objective and scientific evaluation indicator system, the construction of which follows the following principles.

Principle of scientificity. The selection of evaluation indicators should be based on scientific theories and rigorous data analysis to ensure the scientificity and reliability of the indicators. The evaluation indicators should be in line with the actual situation of urban tourism competitiveness, reflecting its influencing factors and intrinsic relationships. Overall, the importance of the indicators should be clear, avoiding subjective assessment as much as possible and fully reflecting the objectives of the evaluation.

Principle of representativeness. Evaluation indicators should be representative and able to represent the level of urban tourism competitiveness. The selection of evaluation indicators should take into account their scope of application and target groups, and be able to provide useful information and guidance while taking into account the realistic background of the indicators and the mutual independence of the indicators, and avoiding duplication.

Principle of operationalization. The selection of evaluation indicators should be operational, i.e., capable of being measured, analyzed, and improved. Evaluation indicators should have clear definitions and methods of access, and be able to be operationalized and managed in practice to facilitate monitoring and tracking of the achievement of objectives.

Principle of quantitative comparability. Evaluation indicators should be quantitative and comparable to facilitate comparison and analysis of urban tourism competitiveness in different regions and time periods. The selection of evaluation indicators should be based on reliable data sources and statistical methods to ensure the comparability and reliability of evaluation results.

According to the principles of scientific, representative, operable, and quantitatively comparable indicator design, the finalized evaluation system includes 4 first-level indicators and 26 s-level indicators for tourism resources, tourism performance, tourism environment, and tourism economy (as shown in Table 1).

**Table 1:** Competitiveness evaluation indicator system of urban tourism.

Level 1 indicators	Level 2 indicators
B1	C₁ Number of A-rated scenic spots
	C₂ Number of scenic spots above level 4A
	C₃ Number of national key scenic spots
	C₄ Number of national cultural heritages
	C₅ Number of star hotels
	C₆ Number of travel agencies
	C₇ Number of tourism distribution centers
	C₈ Number of national model and pilot pedestrian streets
B2	C₉ Gross tourism receipts
	C₁₀ Gross tourism receipts as a share of GDP
	C₁₁ Total number of tourist arrivals
	C₁₂ Average annual growth rate of tourists in the last 5 years
	C₁₃ *Per capita* tourist spending in the last 5 years
B3	C₁₄ Annual passenger traffic for society as a whole
	G₁₅ Air passenger throughput
	C₁₆ Number of urban bus routes
	C₁₇ Total length of urban metro runs
	C₁₈ Total telecommunications services
	C₁₉ 5G base stations per 10,000 people
	C₂₀ Parkland *per capita*
	C₂₁ Number of days with good or better air quality
	C₂₂ sewage treatment rate
B4	C₂₃ Total annual urban GDP
	C₂₄ annual GDP *per capita*
	C₂₅ Share of tertiary sector
	C₂₆ Retail sales of social consumer goods

The indicators for the Level 1 indicators are as described below: B1 Tourism Resources. B2 Tourism performance. B3 Tourism environment.

### 4.3 Evaluation model construction

#### 4.3.1 Random forest empowerment

The basic idea of random forest assignment is to determine the indicator weights by assessing the degree of importance of the indicators. Compared with objective weighting methods such as entropy weighting, it can dig deeper into the relationship between indicators and threat values in the target threat database that already has target threat values, and obtain more reasonable indicator weights. The degree of importance of indicators can be assessed from two aspects.

On the one hand, in the process of modeling, if an indicator makes a greater contribution to the growth process of the regression tree in a random forest, it means that this indicator is more important and should be given a greater weight, which is recorded as α. It is calculated as follows:

Step 1 Calculate the mean square error for each node in each tree, where the mean square error of node *s* is Equation 4:


(4) $$MSE_{s}=\frac{1}{T_{s}}\overset{T_{s}}{\underset{x=1}{\sum }}{\left(c_{s}-j_{x}\right)}^{2}$$

Step 2 Calculate the node contribution of each metric by expressing the contribution of metric y at node s as the difference in the mean square error between the parent and child nodes after branching in Equation 5:


(5) $$Q_{ys}^{MSE}=MSE_{s}-MSE_{l}-MSE_{r}$$

Step 3 Compute the total impact of each indicator by establishing the cumulative effect of indicator y as Equation 6:


(6) $$Q_{ny}^{MSE}=\underset{s\in W}{\sum }Q_{ys}^{MSE}$$

Where W is the set of nodes where indicator y appears in the tth regression tree.

Step 4 Get the weight of each indicator as Equation 7:


(7) $$\alpha_{y}=\frac{\sum_{n=1}^{N}Q_{ny}^{MSE}}{\sum_{y\in w}\sum_{n=1}^{N}Q_{ny}^{MSE}}$$

Where n is the number of regression trees in the forest. w is the set of splitting indicators.

On the other hand, from the results of the model, when the random forest model is trained, we take a method to evaluate the importance of each indicator. This is done by randomly resetting the value of each indicator in the dataset in turn, and then observing the change in model performance. If the model effect drops a lot, this indicates that the indicator has a significant role in influencing the target value. In this case, it is logical to assign a greater weight to this indicator, noting this weight as *β* in Equation 8.


(8) $$R^{2}=1-\frac{\sum_{x=1}^{T}{\left(f_{x}-j_{x}\right)}^{2}}{\sum_{x=1}^{T}{\left(j_{x}-j\right)}^{2}}$$

Where: $f_{x}$ is the model regression value. $j_{x}$ is the true value of sample x . j is the average of the true values of the samples.

Step 1 For the trained Random Forest model choose $R^{2}$ as a measure of model performance, calculated as Equation 9:


(9) $$R_{y}=R_{l}^{2}-R_{e}^{2}$$

Where: $R_{l}^{2}$ is the performance score before reset. $R_{e}^{2}$ is the performance score after reset.

Step 2 Iterate through all the indicators to get the importance score of each indicator. Then the weight of indicator y is Equation 10:


(10) $$\beta_{y}=\frac{R_{y}}{\sum_{x=1}^{w^{\prime }}R_{y}}$$

Where: $w^{\prime }$ is the number of indicators. $R_{y}$ is the indicator importance score.

Finally, α and β are weighted and combined to obtain the indicator weights as in Equation 11:


(11) $$\lambda_{y}=\tilde{\varphi }\alpha_{y}+\left(1-\tilde{\varphi }\right)\beta_{y}$$

Where: $\tilde{\varphi }$ is the adjustment coefficient, taking the value from 0 to 1.

Random Forest empowerment.

#### 4.3.2 Topsis assessment

Topsis is a model commonly used in risk assessment, which is a ranking method that approximates the ideal solution for multi-attribute decision-making problems. The ranking is usually obtained by calculating the distance between each evaluated solution the optimal ideal solution and the worst ideal solution.

Step 1 Determine the threat affiliation matrix $K={\left(k_{xy}\right)}_{w\times t}$ through the affiliation function.

Step 2 Determine the ideal optimal solution $k^{+}=\left(k_{1}^{+},k_{2}^{+},\cdots ,k_{t}^{+}\right)$ and the ideal worst solution $k^{-}=\left(k_{1}^{-},k_{2}^{-},\cdots ,k_{t}^{-}\right)$ .

Step 3 Calculate the distance $D_{x}^{+}$ and $D_{x}^{-}$ of each objective from the ideal optimal solution and ideal worst solution, respectively in Equations 12, 13:


(12) $$D_{x}^{+}=\sqrt{\overset{t}{\underset{y=1}{\sum }}\lambda_{y}{\left(k_{y}^{+}-k_{xy}\right)}^{2}}$$

(13) $$D_{x}^{-}=\sqrt{\overset{t}{\underset{y=1}{\sum }}\lambda_{y}{\left(k_{y}^{-}-k_{xy}\right)}^{2}}$$

Where: $\lambda_{y}$ is the weight of the *y*th indicator, which is obtained from the proposed random forest based assignment method.

Step 4 Get the relative closeness as in Equation 14:


(14) $$S_{x}=\frac{D_{x}^{-}}{D_{x}^{+}+D_{x}^{-}}$$

The relative closeness $S_{x}$ reflects the degree of the trend of each evaluation close to the positive ideal solution, and the larger its value represents that the evaluation object is closer to the optimal ideal solution, and the better the evaluation result is. In this paper, we judge the comprehensive competitiveness of the city and the competitiveness level of each dimension according to the size of Si value, and make a comparative analysis.

**Figure 5:**
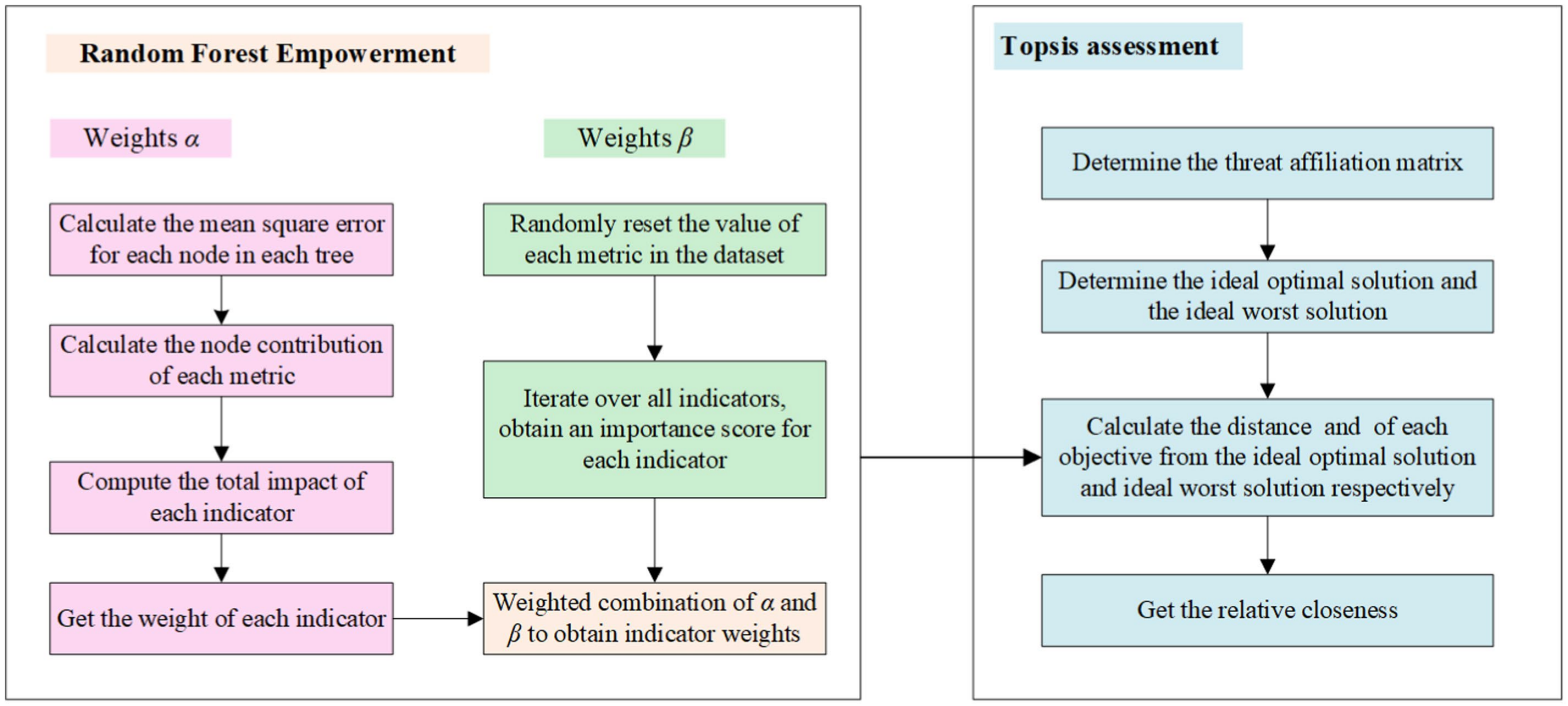
The framework diagram of the evaluation model construction.

The framework diagram of the evaluation model construction method in this paper is shown in Figure 5.

The limitations and assumptions in the overall model are shown below:

Limitations in the model analysis process: random forest assignment determines weights by mining the relationship between indicators and target threat values. Its effectiveness is limited by the quality and completeness of the data and the ability to handle strongly correlated or redundant features in a high dimensional space. The Topsis method is sensitive to the distribution of the original data, especially when the data is extremely skewed, and a simple distance metric may not fully reflect the true strengths and weaknesses of the evaluation target.

Assumptions in the model analysis process: (1) In generating the decision tree, Random Forest uses Bootstrap sampling to generate the training set, assuming that such sampling is representative of the overall population and does not lead to misestimation of the importance of features due to sampling bias. (2) The Topsis method assumes that the decision maker’s preferences are consistent across all the metrics. (3) The Topsis method assumes that the effect between different metrics on the total effect are linear and independent.

### 4.4 The public health implications of this paper’s model

This paper proposes a model for evaluating the competitiveness of urban tourism that is closely related to public health and urban planning practitioners. The model provides a comprehensive framework that integrates the concept of sustainable development into urban tourism evaluation and contributes to the theoretical development on sustainable tourism research. The application of machine learning techniques provides new ways to analyze complex datasets, enhancing our understanding of the multifaceted relationships that affect tourism competitiveness.

From a public health perspective, the model recognizes that environmental and social sustainability are key components of urban tourism competitiveness. This is consistent with the increasingly recognized links between tourism activities, health outcomes and community well-being. By incorporating indicators that reflect the health and quality of life of local residents, the model supports the development of tourism strategies that favor healthier urban environments.

For urban planning practitioners, the model provides a practical tool for assessing the impact of tourism development on urban sustainability. It can guide the decision-making process by providing insights on how to optimize different aspects of urban planning (e.g., transportation, infrastructure, and land use) for sustainable tourism growth. This can lead to the creation of urban spaces that are not only attractive to tourists, but also environmentally sustainable and socially inclusive.

In conclusion, the model presented in this paper addresses the multifaceted challenges of urban tourism development in the context of sustainable development, providing a holistic approach that is highly relevant to public health and urban planning practitioners. By considering the interconnections between tourism, environment, society and economy, the model supports sustainable development that contributes to urban tourism.

By addressing these revision requests, the authors will significantly strengthen their manuscript, making a compelling case for its contribution to the ongoing discourse on sustainable urban tourism development and its implications for public health.

## 5 Result analysis and discussion

### 5.1 Data sources

The cross-section data is characterized by high dispersion and highlights differences, accordingly, the cross-section data of 2020 is adopted to analyze the competitiveness level. The data are mainly obtained from local statistical yearbooks, statistical bulletins on economic and social development of cities, government work reports, websites of the Culture and Tourism Development Commission, etc.

However, there are a number of biases or limitations that may be encountered during the collection and analysis of data that may have an impact on the general applicability of the study’s findings.

First, the timeliness of the data is an important consideration. Due to the limitation of the frequency of data updates, the data used in the model may not reflect the latest tourism development, which will affect the model’s ability to predict current and future tourism competitiveness.

Second, the statistical indicators on which the model relies may not fully cover all the factors that affect the competitiveness of urban tourism. For example, some non-material factors, such as city image and cultural atmosphere, may be difficult to measure through statistical data. These factors may have an important impact on tourism competitiveness but fail to be included in the model.

In addition, there may be some potential biases in the analysis process. For example, the modeling may be based on certain assumptions which may not be consistent with the actual situation.

### 5.2 Optimal model selection

**Figure 6:**
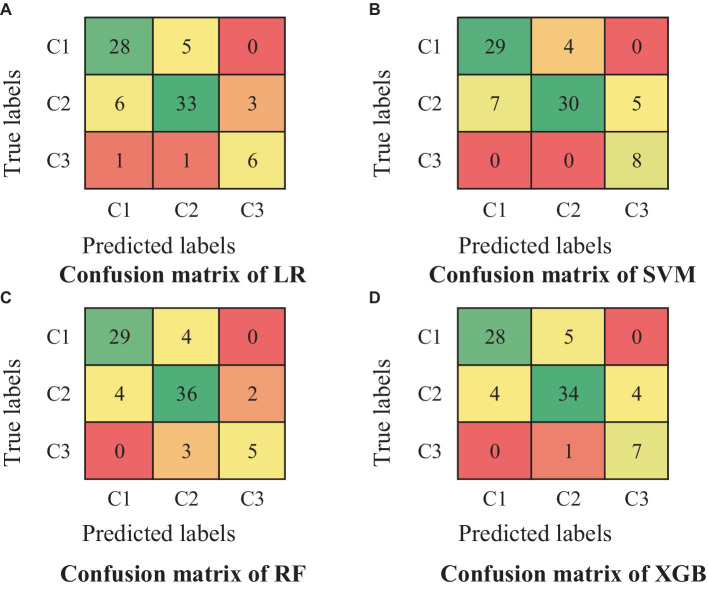
Confusion matrix for prediction of each model.

**Figure 7:**
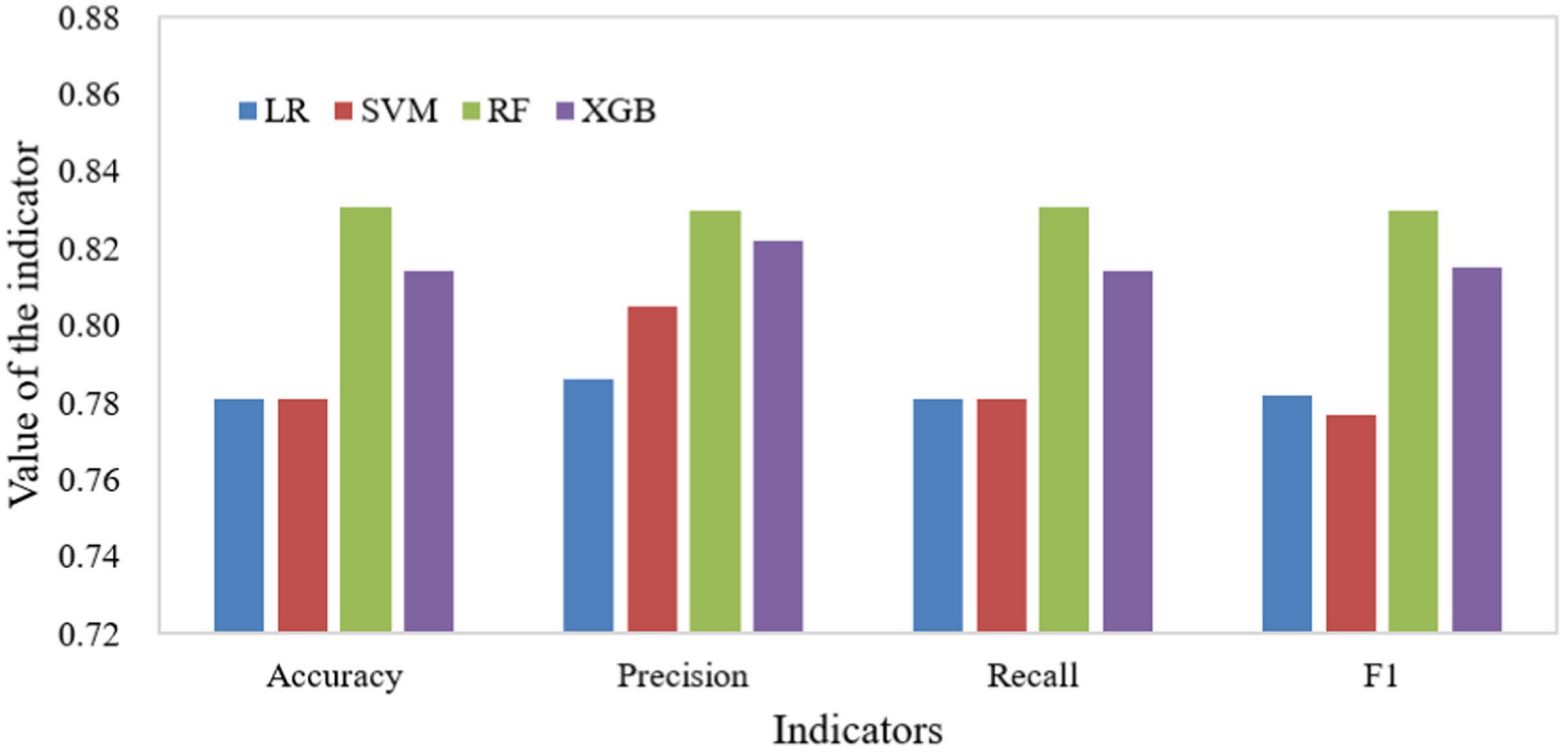
Results of the accuracy assessment metrics for each model.

The dataset is divided into training set and test set in 7:3 ratio. Among them, the training set is used for the creation and training of each machine learning model, and Bayesian optimization is used for the hyper-parameter tuning of the model. The test set is used for model accuracy evaluation of each model, and the confusion matrix (As shown in Figure 6), precision, accuracy, recall, and F1 scores (As shown in Figure 7) of each model are output according to the macro-averaging calculation method.

In the results of the accuracy evaluation of each model, the highest value of accuracy was predicted by RF (0.831), followed by XGB (0.814). Whereas, the lowest value of accuracy (0.781) occurs for the prediction of LR and SVM. The range of values for precision is 0.786 to 0.830 and the highest and lowest values are for the prediction of RF and LR, respectively. RF has the highest recall of 0.831. Whereas LR and SVM have the lowest recall of 0.781. The highest F1 score is for RF (0.830) followed by XGB (0.815) and the lowest value is for SVM (0.777).

Integrating each accuracy evaluation criterion, the evaluation accuracy of the four machine learning models is better overall. Among them, two nonlinear integrated learning models (RF, XGB) have relatively higher recognition accuracy for urban tourism competitiveness, while linear models LR and SVM (linear kernel) have relatively lower recognition accuracy. RF demonstrates superior performance in terms of recognition when compared to the other models. Consequently, it stands out as the most suitable choice for the assessment of urban tourism competitiveness within the context of this study.

### 5.3 Evaluation of urban tourism competitiveness in different provinces

**Figure 8:**
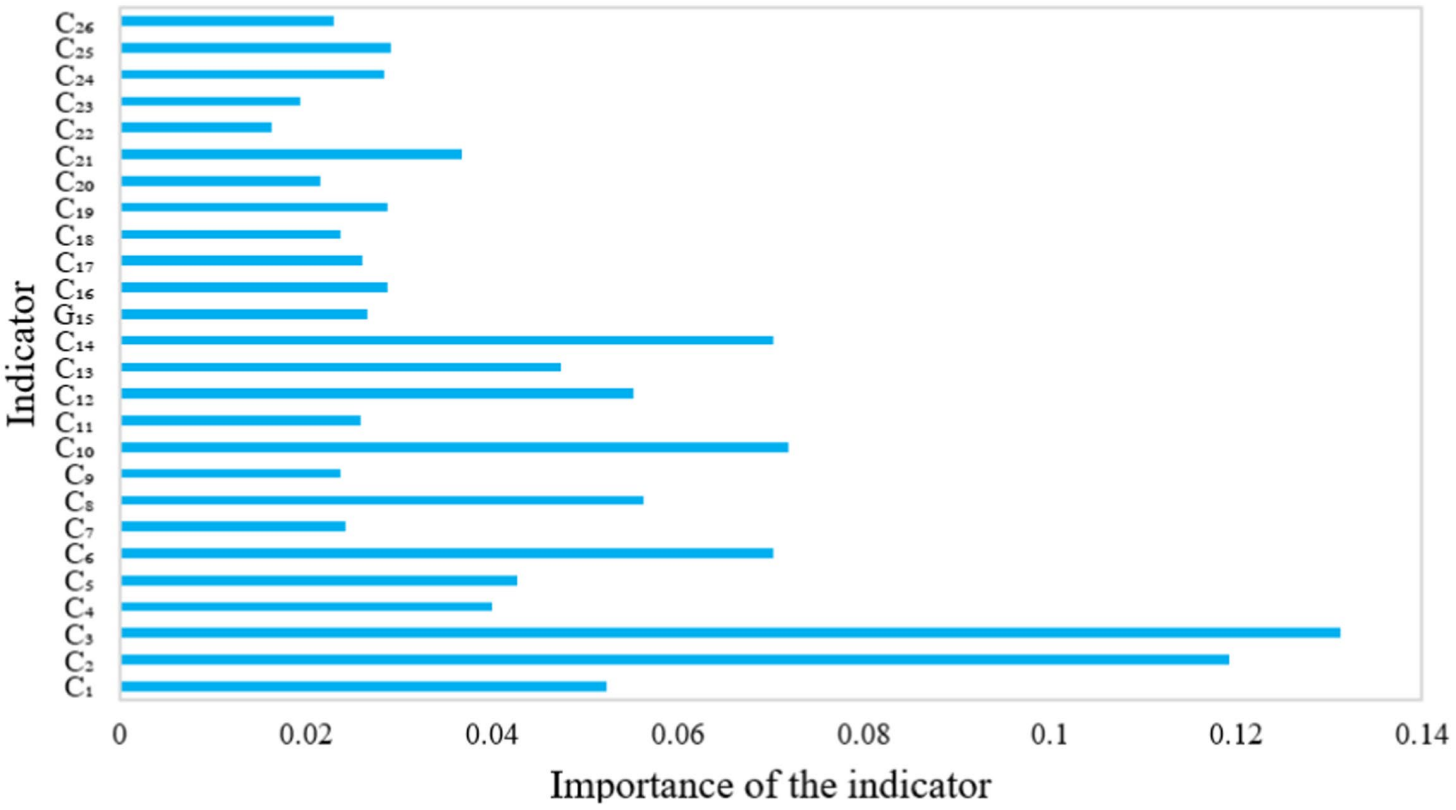
Level of importance of indicators.

Machine learning was used to analyze the importance of indicators and the results are shown in Figure 8.

**Figure 9:**
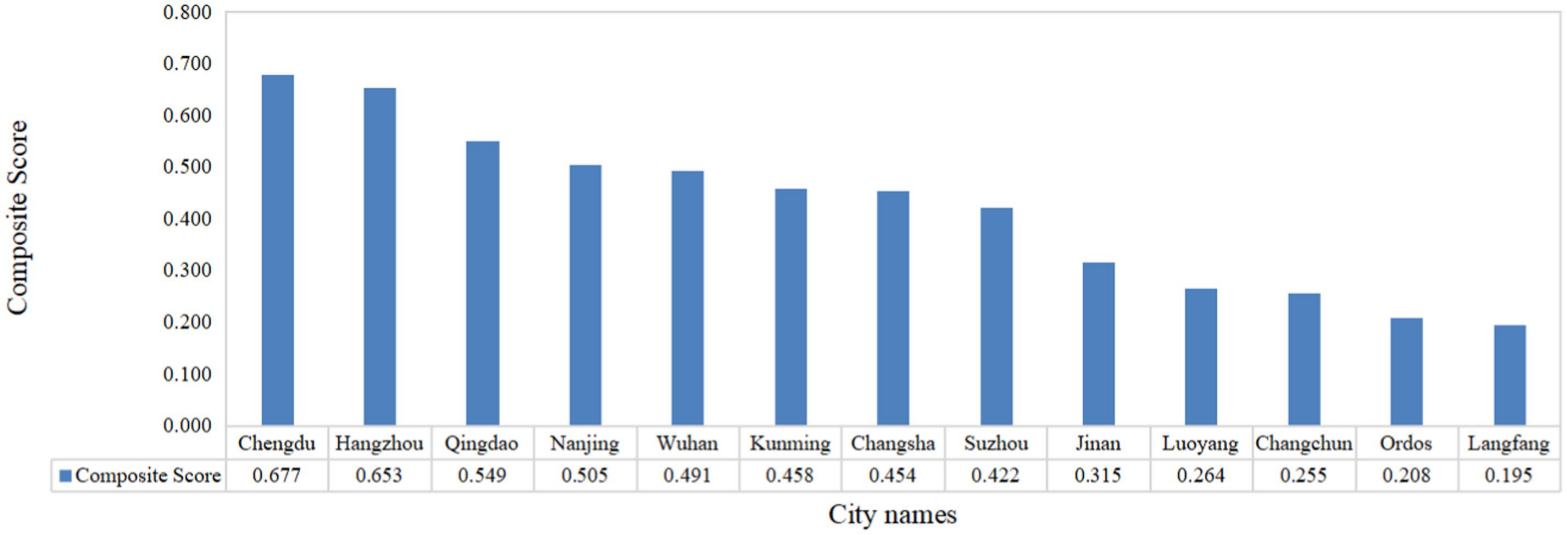
Urban tourism competitiveness composite score.

As can be seen from the composite scores of tourism consumption competitiveness of the 13 demonstration cities in Figure 9, Chengdu ranks first, with Hangzhou, Qingdao, and Nanjing ranking in decreasing order. On the whole, the tourism competitiveness of each city varies greatly and is unbalanced.

This result not only validates the validity of the model but also reveals the opportunities and challenges faced by different cities on the path to sustainable development of the tourism industry. Relating these results to the research objectives, it can be seen that the evaluation model proposed in this paper provides a substantial contribution to the field of public health by helping to identify and prioritize key issues that directly affect public health and environmental sustainability in tourism destinations. By increasing attention to public health indicators in the evaluation of tourism competitiveness, urban decision-makers can be prompted to pay more attention to environmental protection and residents’ health when formulating tourism policies, thus promoting synergistic progress in tourism, public health, and urban environmental development, and realizing sustainable tourism development in the true sense of the word.

### 5.4 Evaluation of tourism competitiveness of different cities within the same province

**Figure 10:**
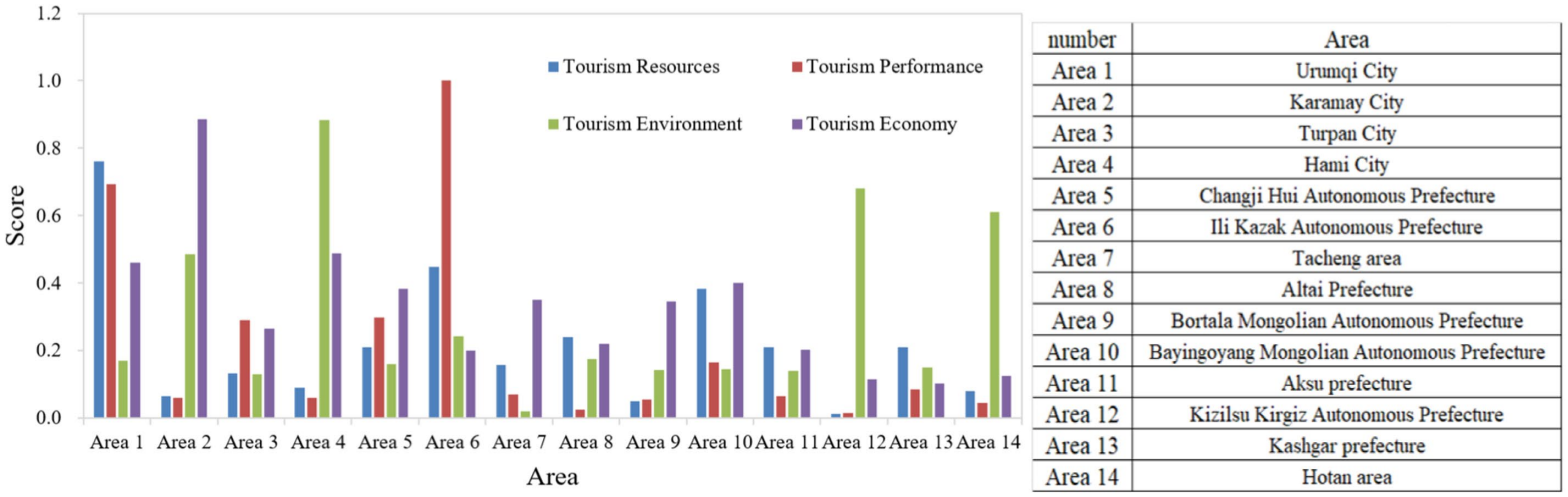
Detailed evaluation results of tourism competitiveness.

The methodology of this paper is used to calculate the tourism competitiveness of the 14 prefectures and cities in Xinjiang, and to obtain the corresponding scores and rankings. Among them, Ili and Urumqi are in the lead (Table 2). The development level of each region in terms of tourism performance, tourism resources, economic support and environmental support of tourism destinations is further calculated (Figure 10).

**Table 2:** Results of tourism competitiveness evaluation.

Level	Scoring range	Area
Strong	[1, 0.5]	Urumqi City
		Ili Kazak Autonomous Prefecture
Relatively strong	[0.5, 0.25]	Hami City
		Changji Hui Autonomous Prefecture
		Karamay City
		Bayingoleng Mongolian Autonomous Prefecture
Weaker	[0.25, 0]	Turpan City
		Kizilsu Kirgiz Autonomous Prefecture
		Hotan area
		Tacheng area
		Altai Prefecture
		Kashgar prefecture
		Kizilsu Kirgiz Autonomous Prefecture
		Bortala Mongolian Autonomous Prefecture

According to the results in Figure 10, the 14 prefectural and municipal cities in Xinjiang can be categorized into three types according to their tourism competitiveness: strong, stronger and weaker. The comprehensive score index of tourism competitiveness ≥0.5 is strong tourism competitiveness, and there are Urumqi City and Ili Kazakh Autonomous Prefecture. Comprehensive score index of tourism competitiveness ≥0.25 and < 0.5 is strong tourism competitiveness, and there are four places: Hami City, Changji Hui Autonomous Prefecture, Kelamayi City, and Bayin’guoleng Mongol Autonomous Prefecture. There are eight places in Turpan City, Kizilsu and Kirghiz Autonomous Prefecture, Hotan, Tacheng, Altay, Kashgar, Aksu and Bortala Mongol Autonomous Prefecture, where the tourism competitiveness index is <0.25, which is considered to be weaker.

As can be seen from Figure 10, in the first level (strong), there are two places, Urumqi and Ili Kazakh Autonomous Prefecture. They are ranked first and second in terms of profitability and tourism resources. Although Ili Kazakh Autonomous Prefecture does not rank high in the economic support and environmental support assessment rankings, it still ranks first in tourism competitiveness in the XUAR based on tourism resources and profitability. The situation is similar in Urumqi.

According to the results of the tourism competitiveness classification of the 14 prefectural and municipal cities in Xinjiang in Table 2 and Figure 10, we can see that there are significant hierarchical differences in the development and comprehensive competitiveness of the tourism industry in different regions. This classification is of great significance for the study of optimization of tourism industry layout, rational allocation of resources, and sustainable development path in Xinjiang, and also provides a scientific basis for local governments to formulate targeted tourism policies and strategies.

The stronger tourism competitiveness of places such as Urumqi and Ili Kazakh Autonomous Prefecture, due to their higher tourism composite scores, suggests that these regions have better tourism environments, infrastructure, service quality, and economic benefits. However, it also implies greater tourist flows and activity intensity. This places higher demands on public health management and environmental protection.

For Hami City, Changji Hui Autonomous Prefecture, Karamay City, and Bayinguoleng Mongol Autonomous Prefecture, which have stronger tourism competitiveness, the process of enhancing tourism competitiveness requires attention to the impact of tourism development on public health and environmental capacity, in addition to continuing to optimize tourism products and services. Through rational planning and management, possible public health problems should be prevented and the positive contribution of tourism activities to the local ecological environment and people’s health should be ensured.

As for regions with weaker tourism competitiveness, such as Turpan City, Kizilsu Kyrgyz Autonomous Prefecture, Hotan Region, Tacheng Region, Altay Region, Kashgar Region, Aksu Region, and Bortala Mongol Autonomous Prefecture, although there is still room for improvement in the current competitiveness of tourism, this at the same time signals great potential for development. In the process of planning and promoting the upgrading of the tourism industry in these regions, lessons should be learned from the successes and failures of other regions, and at the same time, efforts should be made to strengthen the construction of the public health system, and public health standards and requirements should be integrated into the tourism development plan in a forward-looking manner, to achieve synergy between the tourism industry and public health. This will not only enhance the competitiveness of tourism but also protect the health and safety of residents and tourists, as well as the protection of the ecological environment. In addition, the development of tourism can also be used as an opportunity to promote the construction and improvement of public health facilities in these areas, further enhancing the health protection level of residents and tourists.

## 6 Conclusions and recommendations

### 6.1 Conclusion

The tourism business is extremely competitive at the moment, and the degree of standardization of tourist locations is significant. In many places, creating unique tourism brands has become a crucial strategy for increasing tourism competitiveness and drawing in foreign visitors. The importance of growing the tourism industry and its strategic role in regional economic growth have grown even more in the context of sustainable development. It is essential that the tourism business undergo reform and modernization. Within the framework of regional tourism, the concept of combining all parties’ resources to create a travel-integrated destination is congruent with the growth of the tourism industry, enhancing its competitiveness, forging unique brands, and promoting tourism-related side businesses. First, this study builds a city tourist competitiveness evaluation system with 26 specific indicators from four dimensions: tourism economy, tourism consumption performance, tourism resources, and tourism environment. This is based on research on tourism competitiveness evaluation systems. Second, this paper suggests a way to assess the competitiveness of urban tourism by fusing Topsis with machine learning. This method fully explores the wealth of information found in tourism data and effectively avoids the subjectivity of the conventional approach, offering a more thorough and scientific assessment of the competitiveness of urban tourism. This paper’s model, which offers a novel methodology and point of reference for future research on tourism competitiveness, analyzes and measures the ranking of urban tourism competitiveness of different provinces as well as the evaluation of urban tourism competitiveness among various cities and municipalities in the same province.

This study shows that its contribution to environmental health and public well-being lies in the fact that by promoting the evolution of tourism in a more sustainable direction, tourism resources can be more effectively protected and rationally utilized, thus maintaining a healthy ecological environment; at the same time, this move also improves the public’s access to high-quality tourism services, which indirectly improves the public’s experience of quality of life and sense of well-being. The future research directions of this paper are:

1. further explore the applicability of the model in different urban environments, such as analyzing the impact of city size, geographic location, resource endowment, and other factors on the model’s effect, and studying how to adjust and optimize the model parameters according to the specific conditions of different cities.
2. explore the scalability of the model, and study how to apply and validate the model in the evaluation of tourism competitiveness in a wider range of datasets, more evaluation dimensions, and at different levels (e.g., national, regional, scenic spots, etc.).
3. study in depth the dynamic relationship between tourism competitiveness enhancement and environmental health and public welfare, to provide more solid theoretical support and empirical evidence for the formulation of tourism development strategies with both economic benefits and environmental and social benefits.
4. Integrate the results of the model with the planning and management practices of tourism destinations to guide the formulation and implementation of policies, to promote the development of the tourism industry while effectively safeguarding and improving environmental quality and public welfare.

### 6.2 Recommendations for sustainable urban tourism

Based on the above conclusions, to realize the sustainable development of urban tourism, suggestions can be made in the following aspects:

1. Optimize the development and protection of tourism resources: cities should pay attention to the protection and rational development of tourism resources to ensure their sustainable use. They should carry out in-depth excavation of tourism resources with characteristics such as natural landscapes and historical and cultural relics to enhance their tourism value, and take effective measures to protect these resources to prevent overdevelopment and destruction.
2. Enhance the quality of tourism services: Improve the quality of tourism employees and enhance the level of tourism services, including the service areas of tour guides, accommodation, catering, and transportation. Through the provision of quality services, the satisfaction of tourists will be enhanced and the city’s tourism competitiveness will be improved.
3. Developing characteristic tourism products: according to the city’s characteristics and advantages, develop tourism products with special features, such as folk tourism, ecotourism, red tourism, and so on. Differentiated tourism products, attract different types of tourists and improve the attractiveness of the destination.
4. Strengthen tourism publicity and promotion: Make full use of various media channels to increase the publicity of the destination and improve its visibility. At the same time, strengthen cooperation with travel agencies, online travel platforms, and other partners to broaden tourism marketing channels.
5. Innovate tourism marketing strategies: utilize the Internet, big data, and other modern technological means to analyze tourists’ needs and behaviors, formulate precise marketing strategies, and enhance the attractiveness of the destination.
6. Promote the integration and development of tourism with other industries: the integration and development of tourism with other industries such as agriculture, industry, culture, and sports can enrich the content of tourism products, extend the tourism industry chain, and improve the economic benefits of tourism. For example, the development of rural tourism, industrial tourism, sports tourism, and so on.
7. Improve tourism infrastructure: Strengthen the construction of tourism infrastructure, including transportation, accommodation, catering, scenic spot facilities, etc., to provide tourists with a convenient and comfortable tourism environment.
8. Implement green tourism development strategy: advocate green tourism and promote low-carbon and environmentally friendly tourism methods, such as hiking and cycling. At the same time, strengthen the management of tourism waste disposal, energy saving, and emission reduction to reduce the impact of tourism on the environment.
9. Improve tourism policy support: The government should introduce a series of policies favorable to the development of tourism, such as tax incentives, land policies, financial support, etc., to provide a strong guarantee for the sustainable development of tourism.
10. Establish a sound tourism supervision system: strengthen the supervision of the tourism market, standardize the order of the tourism market, and protect the rights and interests of tourists. At the same time, establish a sound tourism safety supervision system to ensure tourism safety.

Through the above measures, we can enhance the competitiveness of urban tourism and realize the sustainable development of tourism. At the same time, it provides tourists with a better tourism experience and promotes the development of the regional economy.

## Data availability statement

The original contributions presented in the study are included in the article/supplementary material, further inquiries can be directed to the corresponding author.

## Author contributions

JS: Writing – review & editing, Software, Investigation, Formal analysis, Conceptualization. BX: Writing – review & editing, Writing – original draft, Methodology, Data curation, Conceptualization.
